# Several *Nocardia abcessus* bronchiolitis in a patient treated with inhaled corticosteroids: a case report

**DOI:** 10.1186/s13223-023-00779-2

**Published:** 2023-04-03

**Authors:** Estelle Cascarano, Murielle Frappa, Bruno Degano, Isabelle Pelloux, Christel Saint-Raymond, Hubert Gheerbrant

**Affiliations:** 1 Service Hospitalier Universitaire Pneumologie Physiologie, Centre Hospitalier Universitaire Grenoble Alpes, Grenoble, France; 2 Service de Bactériologie et d’Hygiène Hospitalière, Centre Hospitalier Universitaire Grenoble Alpes, Grenoble, France; 3 Service de Pneumologie, Centre Hospitalier Metropole Savoie, Chambéry, France; 4 Paris, France

**Keywords:** Nocardiosis, Asthma, Inhaled corticosteroids, Bronchiolitis, Case report

## Abstract

Nocardiosis is a disease that mainly affects immunocompromised patients. Inhaled corticosteroids (ICS) are standard of care for asthma. This treatment can induce respiratory infections but no case of bronchiolitis nocardiosis have been described so far. A 58-year-old man, with history of controlled moderate allergic asthma, develop an increased cought in the last two years associated with dyspnea on exertion. Within two months, although ICS were increased to high doses, symptoms worsened due to a severe obstructive ventilatory disorder as revealed by pulmonary function tests (PFT). Small-scale lesions (< 10%) were found on chest computed tomography (CT). A bronchoalveolar lavage (BAL) found *Nocardia abcessus*. After six months of Sulfamethoxazole/Trimethoprim, PFT results improved and chest CT became completely normal. We therefore present the case of a bronchiolitis nocardiosis with several bronchial syndrome and the only immunosuppressive factor found were ICS.

## Background

Nocardiosis is an infectious disease caused by gram positives bacilli aerobics of the genus *Nocardia*, an ubiquitous bacteria in the environment. Infected tissues are most often the lung, but also the brain and the skin. Pulmonary nocardiosis occurs most often in patients that are immunocompromised or with severe chronic lung disease. The clinical presentation is a pneumonia persisting after usual treatment. It is a severe infection that requires high-dose of antibiotic, most often by sulfamethoxazole/trimethoprim, over several months [1].

Asthma is a common chronic disease and ICS is the standard treatment [2]. The main complication of ICS is respiratory infection [3].

However, ICS are not known to favour nocardiosis in the absence of another associated immunosuppressive factor.

We share with you the case of a moderate asthmatic patient who developed pulmonary nocardiosis with predominant bronchiolitis damage.

## Case presentation

We assessed a 58-year-old patient who had been treated for moderate allergic asthma since childhood. His treatment included montelukast, a fixed combination of ICS, and a long-acting β2 agonist (fluticasone-salmeterol 250/50 μg two doses per day) via the Diskus powder inhaler. He also had hypertension treated with enalapril and lercanidipine. He worked as a University teacher and was a non-smoker.

His asthma was controlled without the use of oral corticosteroids (OC) until two years ago. In February 2020, symptoms of bronchitis appeared with a productive cough and exercise induced dyspnea without wheezing. The chest X-ray was normal. His symptoms were not improved by several antibiotic courses (pristinamycin, amoxicillin and amoxicillin/clavulanic acid). Having no signs of obstruction, his general practitioner did not prescribe OC and he was referred to the Grenoble Alpes University hospital. He consulted in pneumology in September 2020. He was noted to have a dyspnea rating of 1 on the Medical Research Council Scale with some ronchi but no gastroesophageal reflux. His PFT's highlighted a moderate non-reversible obstructive ventilatory disorder with a forced expiratory volume in one second on forced vital capacity (FEV1/FCV) ratio at 50%, a FEV1 at 2.9L (72% predicted) and a forced expiratory flow at 25–75% (FEF25-75) at 1.6L (48% predicted). On the biological assessment, the specific anti-aspergillary immunoglobin E (IgE) was less than 0.3 kU/L (Immunocap Phadia), the anti-aspergillary serology by immunoelectrophoresis was weakly positive at 2.82 (ELISA), the total IgE was less than 100 KU/L. Eosinophilic polynuclear blood concentration was 0.1 G/L. CRP was less than 3 mg/L. ICS were therefore increased to high doses.

**Fig. 1:**
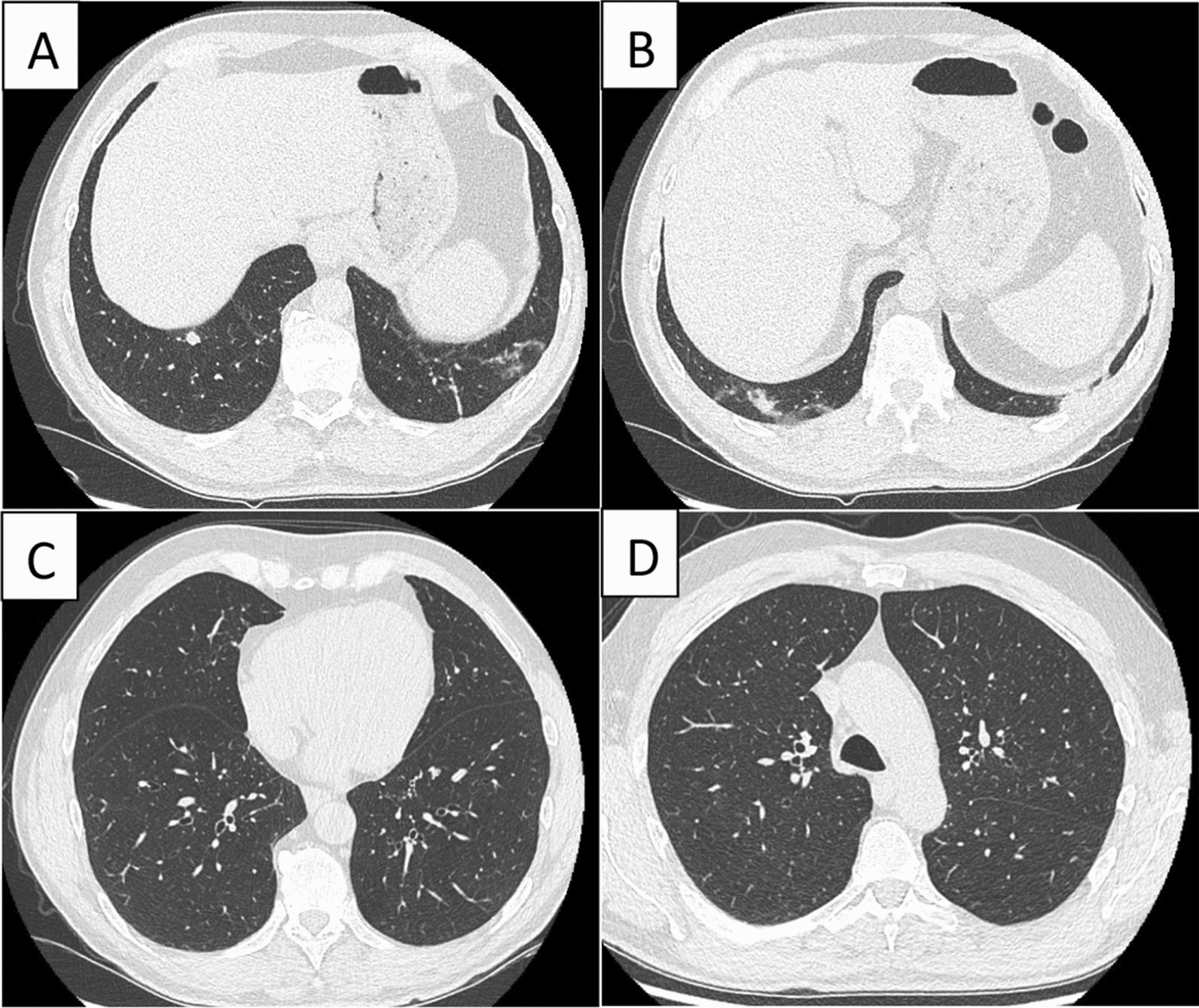
Chest CT scan. **A** right 8 mm juxta-diaphragmatic nodule. **B** right and left basi thoracic bronchiolitis. **C**, **D** Medium and upper sections of the lungs without anomalies

Two months later, the patient felt no improvement of his cough and his dyspnea worsened. FEV1 was at 2.6L (63% predicted) and a FEF25-75 at 1.3 L (38% predicted). On the cardiopulmonary exercise test, he reached a maximal oxygen consumption at 31.2 ml/kg (90% predicted). There was a respiratory limitation with an exhaustion of the ventilatory reserve (9% predicted at peak effort) without desaturation. Cardiovascular adaptation was normal. A chest CT scan revealed foci of basithoracic bronchiolitis adjacent to a right 8 mm juxta-diaphragmatic nodule (Fig. 1A, B). A bronchial fibroscopy were performed and found a normal macroscopic aspect. The cytology of bronchial fluid showed neutrophilic inflammation. The culture of BAL was positive to *Nocardia abcessus* at 10^4^ CFU/mL.

The interrogation didn’t reveal a particular context of contamination (soil, rotten organic matter and waters). He had no history of invasive bronchial procedures, including fibroscopy at the origin of the potential wound. An immunocompromised assessment was performed. The gammaglobulin level was at 12.7 g/L, HIV serology was negative, the neutrophil count and fasting blood glucose were normal. Lymphocyte immunophenotyping was normal with CD4 + T cell and CD8 + T cell count of 0.99 and 0.51 G/L respectively. Anti-GM-CSF antibodies were negative. The brain CT and skin examination were normal.

**Fig. 2:**
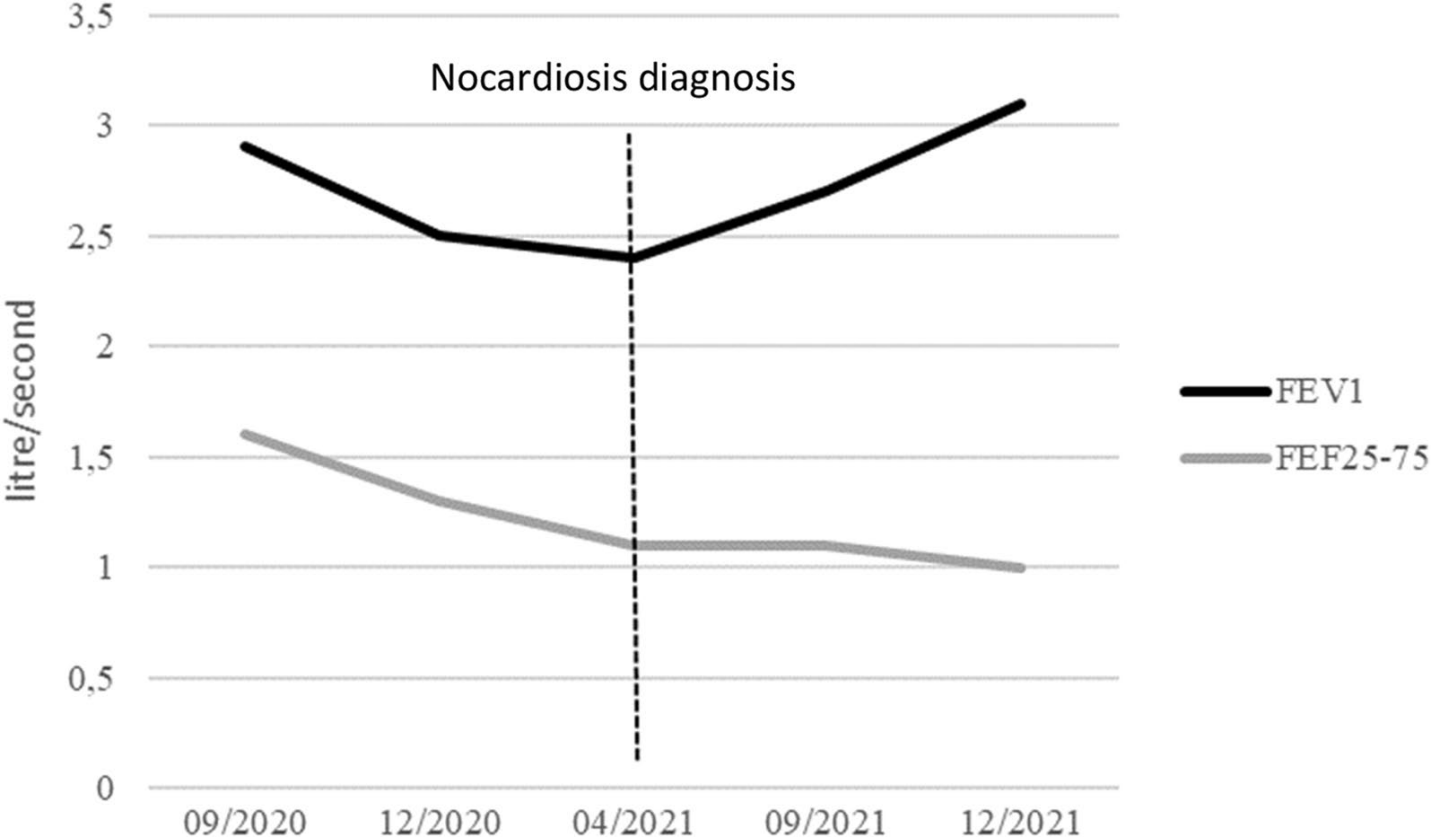
Evolution of FEV1 and FEF25-75 as a function of time before and after nocardiosis diagnosis

Treatment with sulfamethoxazole 800 mg + trimethoprim 160 mg two tablets three times a day was started and ICS were suspended. After three months of antibiotics, the cough and dyspnea decreased without exacerbation of asthma. After six months of treatment, the FEV1 was 3.1L (79% predicted) and the FEF25-75 was stable at 1.1L (32% predicted) (Fig. 2). Chest CT scan had completely normalized.

## Discussion and conclusions

ICS treatment is usually prescribed in asthma. In our case, the patient was receiving a medium dose of ICS. A Japanese team reported a case of nocardiosis caused by *Nocardia otitidiscaviarum* in a 72-year-old patient consuming high dose of fluticasone for asthma [4]. Their patient presented with an acute pneumonia following influenza A infection. The chest CT showed an irregularly shaped solid opacity and a cavitary mass. Pulmonary nocardiosis are most often characterised by acute pneumonitis and pleural effusions with radiological images of lung infiltration and excavated nodules. Cases of pulmonary nocardiosis associated with an ICS treatment have been described in patients without asthma but with a structural lung disease inducing local immunodeficiency: silicosis [5] and COPD [6], but the clinical presentation is that of an acute pneumopathy. In our case report, the infection is chronic, bronchial and the lung parenchyma is preserved. To our knowledge, a nocardiosis bronchiolitis has not been clinically described [7]. However, bronchiolar damage due to a nocardia infection has already been observed on a lung biopsy in context of pneumonia [8].

There is limited data on cases of pulmonary nocardiosis in immunocompetent patients. A case report in 2021 [9] in a 77-year-old non-smoking Japanese patient with no comorbidities found a lung abscess with pleural effusion. There are no other cases in the literature to our knowledge. The need for specific growth medium and longer incubation period of distal lung samples may explain the lack of knowledge about this infection. Nevertheless, this case raises the question of looking for nocardiosis in immunocompetent patients that present a clinical picture of chronic bronchiolitis.

Although ICS is classically considered as a well-tolerated medication, international recommendations insist on the need to lower ICS to the minimum dose to achieve asthma control [2]. Our patient was on moderate-dose ICS while having controlled asthma for 2 years. Therefore, the decrease of ICS doses should always be discussed.

In conclusion, we describe for the first time the case of a nocardia infection with severe bronchiolar damage in a patient whose only immunosuppressive factor was an ICS treatment. His prognosis was favourable after antibiotic treatment. The risk of respiratory infection in a patient treated by ICS should not be overlooked.

## Data Availability

The datasets used and/or analyzed during the current study are available from the corresponding author on any reasonable request.

## Abbreviations

BAL: Bronchoalveolar lavage
COPD: Chronic obstructive pulmonary disease
CT: Computed tomography
ICS: Inhaled corticosteroids
FEF25-75: Forced expiratory flow at 25–75%
FEV1: Forced expiratory volume in one second
FVC: Forced vital capacity
OC: Oral corticosteroids
PFT: Pulmonary function tests
